# Implications for the future of the HIV epidemic if drug resistance against dolutegravir cannot occur in first-line therapy

**DOI:** 10.7448/IAS.18.1.20824

**Published:** 2015-12-04

**Authors:** Mark A Wainberg, Thibault Mesplede

**Affiliations:** McGill University AIDS Centre, Jewish General Hospital, Montreal, QC, Canada

It is remarkable that no case of drug resistance against dolutegravir (DTG) in first-line therapy has yet been described in the scientific literature in spite of the fact that this anti-HIV integrase inhibitor was 1) approved by the Food and Drug Administration (FDA) of the United States in July of 2013 [1] and 2) included in US and European treatment guidelines from the autumn of 2013. We should ask whether this observation is significant and what the implications of having a drug that might be impervious to the development of drug resistance could be on the future of the HIV epidemic.

First, it should be noted that several clinical trials have documented the superiority of triple drug regimens that include an integrase inhibitor over those that are based on use of either a HIV protease inhibitor or a non-nucleoside reverse transcriptase inhibitor (RTI) [2, 3].

Second, it is important to point out that the non-occurrence of drug resistance against DTG in initial therapy also extends to the nucleoside compounds with which it has been co-utilized in a triple drug therapeutic regimen [2–5]. This means that no resistance has been shown to occur in relevant clinical trials against both lamivudine (3TC) and emtricitabine, even though it is well understood that the reverse transcriptase M184V mutation that confers resistance against these two nucleoside drugs is usually the quickest to emerge in the aftermath of HIV treatment failure. Although these clinical trial results have virtually all been obtained in patients infected by HIV-1, some of whom were also co-infected with hepatitis C virus (HCV), there are also data to indicate that DTG is as effective against HIV-2 as against HIV-1 [6, 7]. In addition, rates of treatment success in the above-mentioned trials commonly fell to approximately 80% after 96 weeks of treatment. However, this occurred in the absence of any demonstrable drug resistance, suggesting that approximately 20% of treatment failures were due to non-adherence to therapy.

A third point is that the FDA approval of DTG in 2013 has led to the use of this compound by HIV-positive patients in some of the poorest areas of the United States, including inner-city Washington, DC, the South Bronx, and inner-city Los Angeles. In these areas, rates of co-infection by a variety of co-morbid agents such as HCV are high and levels of adherence to antiretroviral (ARV) drug regimens have been reported to be low [8–10]. These are some of the reasons for which a recent editorial has called for the immediate acceptance of DTG as a cornerstone of HIV therapy in all countries in the world including those in sub-Saharan Africa and other developing country settings [11]. Is this justified? What is the evidence that DTG might be a qualitatively different drug than any other ARV? And might DTG potentially be able to play a key role in regard to helping to end the HIV epidemic?

To start, it is not true that resistance against DTG cannot occur if this drug is not used in first-line therapy. A variety of studies have, in fact, documented that prior failure with either raltegravir (RAL) or elvitegravir (EVG), the first two FDA-approved HIV integrase inhibitors, can compromise future responsiveness to DTG [12, 13]. This is because the mutations that confer resistance to each of the former drugs can also confer cross-resistance against DTG. In contrast, the use of DTG in a previously untreated patient or in an individual who has failed other drugs but never before been treated with an integrase inhibitor may favour the initial selection of mutations at positions R263K or G118R in the integrase coding region [14]. The latter two mutations confer only a very limited degree of resistance against DTG but also have the consequence of greatly diminishing HIV replicative capacity, particularly if a second mutation at a position such as H51Y is also selected [15, 16]. This means that DTG can retain potent antiviral activity despite the presence of these substitutions in integrase at the same time that the viruses that are minimally (and not significantly) resistant to this drug are hugely disadvantaged in regard to replication ability.

This has led to the hypothesis that DTG-resistant variants of HIV will not be detected following first-line therapy in plasma and other body fluids [17], either because resistant variants cannot emerge or are present in such low quantities as to remain undetectable. In addition, the presence of a DTG-selected HIV variant that is incapable of rapid growth and mutagenesis may mean that such viruses remain far more durably susceptible to the host's anti-HIV immune response than would be the case if other drugs were employed.

In fact, several studies have shown that the use of DTG as an initial integrase inhibitor in patients who had previously failed other regimens and who had limited treatment options also favoured the selection of the R263K mutation associated with limited resistance against DTG [14]. What is key, however, is that viral rebound in these cases did not occur to high levels but stayed relatively low, in the range of 1000 to 2000 copies/ml, a finding that might be explained by the negative impact of R263K on viral replication capacity.

The 15th European AIDS Conference (EACS) has now provided additional data in support of the above hypothesis. First, a small 20-patient study termed *PADDLE* (Pilot Antiretroviral Design with DTG/Lamivudine) by Figueroa *et al*. of Buenos Aires, Argentina, demonstrated that the use of only two drugs, DTG plus 3TC, in combination as initial therapy led to profound reductions in viral load over 48 weeks without viral rebound or the development of resistance in all patients tested [18].

Two other studies by Rojas *et al*. and Katlama *et al*. presented at EACS 2015 dealt with the topic of initial suppression of HIV viral load to below 50 copies/ml by traditional ARV chemotherapy followed by maintenance only on DTG monotherapy [19, 20]. It is unclear why 3TC was not also given to these patients. Nonetheless, in both of these studies, representing a total of 61 patients, high viral rebound was observed in three individuals who had previously received either RAL or EVG as a part of prior therapy [20]. This result suggests that resistance mutations that might have arisen in the aftermath of RAL/EVG usage might have compromised the subsequent use of DTG, as has been observed in the VIKING clinical trials [12, 13, 21]. In a fourth patient who had also previously received RAL [19], lower viral rebound was observed alongside the occurrence of a G118R mutation in integrase that is also associated with minimal level DTG resistance and diminished HIV replication [16].

These findings are consistent with predictions that might be made from prior results that drug resistance against DTG in first-line therapy has not occurred over two and one-half years since the initial use of this drug in disadvantaged populations in the United States and more than five years since the first use of DTG in clinical trials conducted on previously drug-naive patients throughout the world.

What now are the implications that we should consider?

First, there should be little doubt that DTG should now form a cornerstone of all current efforts aimed to promote the concept of treatment as prevention. The reasons for this recommendation are as follows:

1. Resistance against DTG has never been reported in clinical trials of first-line therapy (or in settings such as inner-city Washington, DC) more than two years after its approval by the FDA.
2. Resistance against the nucleos(t)ide RTIs with which DTG has been co-utilized has never been reported in the peer-reviewed scientific literature following first-line therapy. These clinical findings are without precedent.
3. The mutations in HIV integrase that are selected by DTG and that confer low-level resistance to it result in greatly diminished viral replication capacity and integrase activity that may be incompatible with viral survival [11].
4. No compensatory mutations that would restore viral replication in the aftermath of DTG resistance have ever been observed.
5. DTG has a very long half-life of activity both within cells and in biochemical assays [22].
6. DTG is one of the safest and best-tolerated drugs in the armamentarium, can be co-formulated and requires only once-daily dosing.
7. DTG has minimal drug-drug interactions with other ARV compounds and with other drugs used to treat HCV as well as unrelated conditions including tuberculosis.

In addition, consideration should be given to the possibility that the non-occurrence of drug resistance against DTG in initial therapy, a finding that is without precedent, may mean that we might actually be able to entertain the possibility of treatment interruption of DTG-based therapy followed by its reintroduction at a future time, without having to worry about the emergence of DTG resistance mutations. Such a possibility could conceivably usher in new ideas aimed at attaining a functional cure of HIV infection, if a means could be found to convert archived wild-type viruses into more attenuated forms that contain the mutations associated with minimal DTG resistance that also impair viral replicative capacity [17].

At the very least, we seem to be moving rapidly toward a recommendation that DTG should be everyone's first choice as a drug to use in initial HIV therapy.
